# The use of prostate MR for targeting prostate biopsies

**DOI:** 10.1259/bjro.20180044

**Published:** 2019-06-19

**Authors:** R. Phelps Kelley, Ronald J. Zagoria, Hao G. Nguyen, Katsuto Shinohara, Antonio C. Westphalen

**Affiliations:** 1 Department of Radiology and Biomedical Imaging, University of California, San Francisco, California; 2 Department of Urology, University of California, San Francisco, California; 3 University of California, San Francisco Helen Diller Family Comprehensive Cancer Center, San Francisco, California

## Abstract

Management of prostate cancer relies heavily on accurate risk stratification obtained through biopsies, which are conventionally performed under transrectal ultrasound (TRUS) guidance. Yet, multiparametric MRI has grown to become an integral part of the care of males with known or suspected prostate cancer. This article will discuss in detail the different MRI-targeted biopsy techniques, their advantages and disadvantages, and the impact they have on patient management.

## Introduction

Prostate cancer is an extremely prevalent and incident cancer in the United States,^1^ but is commonly overdiagnosed: many patients are diagnosed with indolent disease.^2^ One of the consequences of this phenomenon has been a change in the treatment paradigm, with an increasing number of males opting for active surveillance (AS), rather than primary definitive therapy.^3^

Management decisions rely heavily on accurate risk stratification obtained through both initial and repeat systematic biopsies, which are conventionally performed under transrectal ultrasound (TRUS) guidance. Yet, multiparametric MRI has grown to become an integral part of the care of males with known or suspected prostate cancer. This is in large part due to the development of the Prostate Imaging-Reporting and Data System (PI-RADS)^4^ and the advent of MRI-targeted biopsy, both of which allow for more accurate characterization and longitudinal surveillance of prostate cancer.^5,6^ This article will discuss in detail the different MRI-targeted biopsy techniques, their advantages and disadvantages, and the impact they have on patient management.

### Limitations of systematic TRUS-guided biopsy

**Figure 1.**
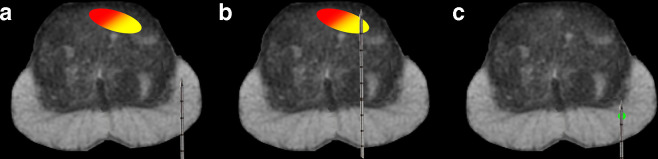
Limitations of TRUS-guided biopsy. Clinically significant tumors may not be detected (a), the tumor grade may be underestimated (b), and clinically insignificant tumors may be overdiagnosed (c). TRUS,transrectal ultrasound.

TRUS-guided biopsies are typically performed using a systematic, extended-sextant approach that samples different regions of the prostate, although with a particular focus on the peripheral zone, in which cancer is more likely to be present.^7^ While it is still considered by many as the gold standard, systematic TRUS-guided biopsies have important limitations (Figure 1). First, they may either miss entirely or undergrade clinically significant prostate cancer (underdiagnosis) in up to 30% of cases. Second, they may detect isolated Gleason score (GS) 3 + 3 disease, which is considered clinically insignificant prostate cancer (overdiagnosis).^8^

While overdiagnosis is essentially unavoidable in random sampling, underdiagnosis arises, at least in part, from a desire to minimize the number of cores obtained while sufficiently sampling the gland. This has led to the recommendation of multiple sampling schemes, which—although improved over time—are still prone to missing cancers, in particular anterior and apical tumors in the setting of pronounced prostatomegaly.^7,8^

By contrast, undergrading of prostate cancer arises from the random chance that a core biopsy may sample part of a cancer, but not necessarily its most aggressive cells. Multiple studies have demonstrated that conventional TRUS-guided biopsies commonly underestimate the GS of prostate cancer. Berglund et al, found that 27% of males thought to have low-risk disease were upgraded upon immediate systematic resampling.^9^ This is in line with a host of other studies, with a reported range of 2.5–28% for upgrading disease on confirmatory re-biopsy.^10^ Further, this rate of upgrade was achieved only through repeat non-targeted systematic sampling. Using data from elective prostatectomies, which provide a truer measure of sampling error, El Hajj et al found that 50% of males thought to be low-risk in fact had higher-grade cancer and were ineligible for AS.^11^ Additional large studies have shown supporting data that systemic biopsies undergrade cancer *vs* prostatectomy in 30–43% of cases,^12–14^ with Kvåle et al highlighting that more than 40% of GS ≤6 biopsies were upgraded to GS ≥7 at prostatectomy.^12^ There are two solutions to reduce undergrading: increase the number of systematic cores;^15,16^ or target higher-yield lesions, as made possible by MRI.

### Accuracy of MRI

Until the publication of PI-RADS, acquisition protocols, interpretation, and reporting were not standardized, and the data describing the accuracy of MRI was heterogeneous and difficult to compile.^17,18^ While variations persist,^19,20^ the data that reflects the PI-RADS guidelines is more robust. Using this guideline, multiparametric MRI has a reported sensitivity and specificity of 85 and 71% for any prostate cancer,^21^ and 87 and 45% for GS ≥3 +4 disease, generally considered to represent clinically significant prostate cancer.^20^ PROMIS, a large study that investigated the use of MRI prior to an initial biopsy for suspected prostate cancer, but used a Likert scale instead of PI-RADS, found similar results (sensitivity = 93%, specificity = 41%).^22^ The negative predictive value of PI-RADS for clinically significant prostate cancer is high, with reported values of 83%, 91.7%, and 98.0%.^23–25^ When a prostate cancer is not detectable with MRI, it is generally lower grade (GS ≤6), a satellite lesion, or apical in location.^26^

### MRI-targeted biopsy techniques

Multiparametric MRI is used to identify lesions that likely represent high-grade prostate cancer. Once identified, these lesions can then be targeted using one of three strategies: (1) TRUS-guided cognitive MRI targeting, (2) MRI-TRUS fusion targeting, and (3) in-bore MRI targeting.

### TRUS-guided cognitive MRI targeting

With cognitive MRI targeting, a provider performs a conventional systematic TRUS-guided biopsy, but also attempts to mentally map the location of the target lesion identified on MRI to the real-time TRUS images. As it requires no specialized equipment aside from the usual ultrasound scanner and transducer, TRUS-guided cognitive MRI targeting is the cheapest form of MRI targeting, and is widely available to patients.

**Figure 2.**
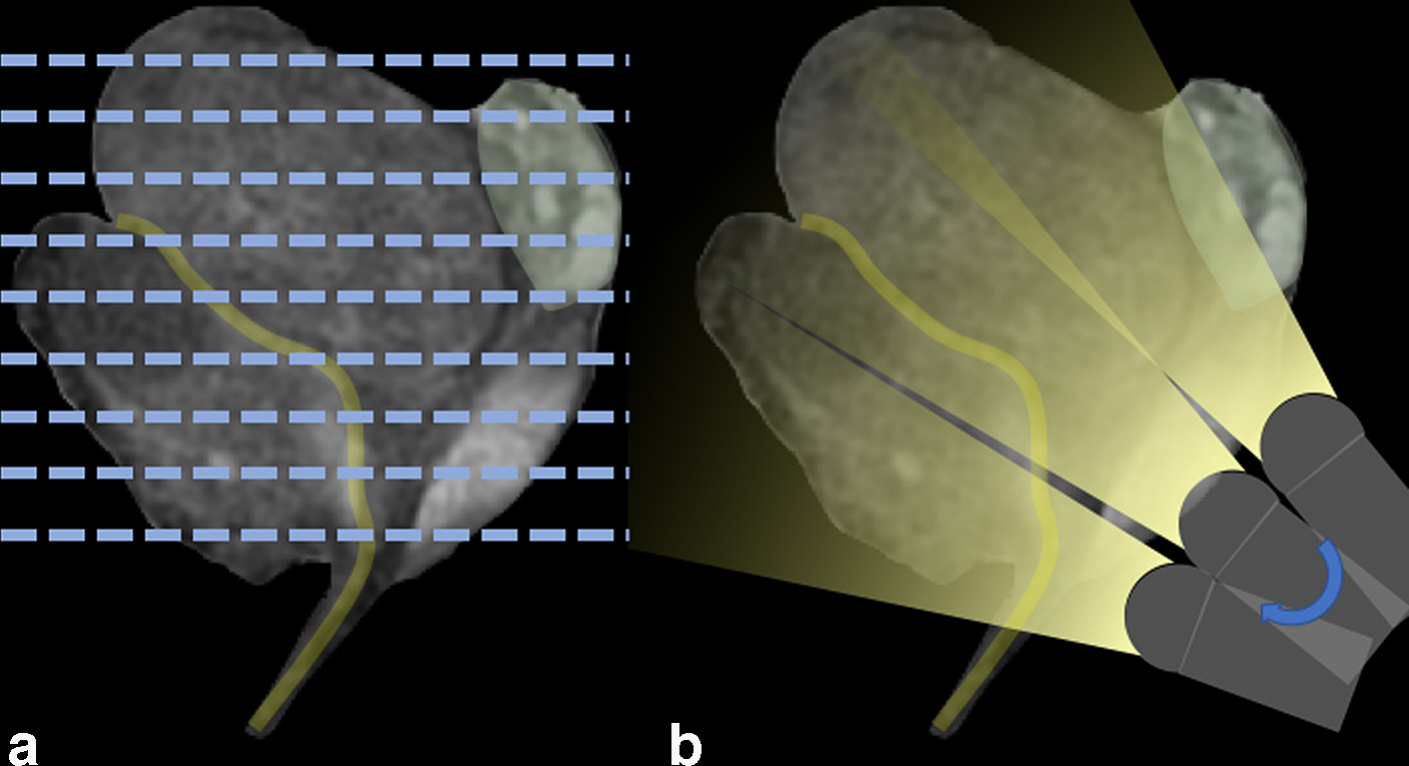
MRI and TRUS images are obtained with different obliquity, a factor that must be taken into consideration when considering the reported location of lesions during biopsies performed under cognitive co-registration. TRUS,transrectal ultrasound.

**Figure 3.**
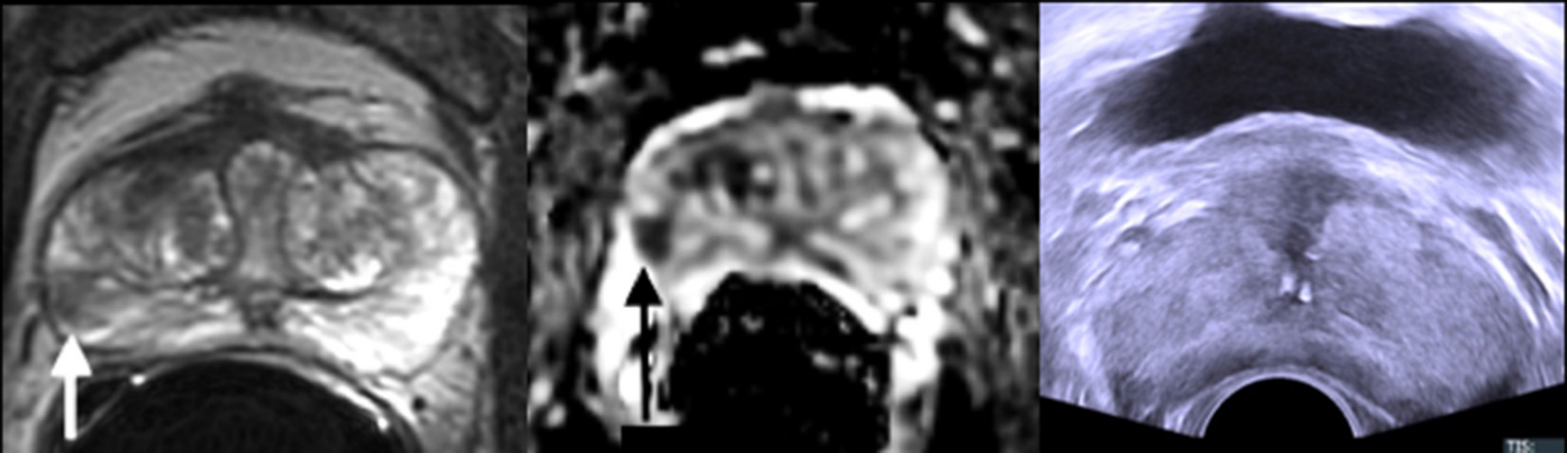
A lesion in the right mid peripheral zone demonstrates T2 hypointensity (a) and a very low ADC (b), compatible with a PI-RADS score of 4. Nevertheless, TRUS in the same patient demonstrates no clear hypoechoic correlate (c), leaving the operator to estimate the target position on the ultrasound image, which may be difficult in a homogeneous gland. This target was shown to represent GS 4 + 4 cancer on prostatectomy. ADC,apparent diffusion co-efficient; AS, active surveillance; GS, Gleason score; TRUS,transrectal ultrasound.

The cognitive co-registration of TRUS and MRI can at times be challenging, and its accuracy is to a great extent reliant on the skill and experience of the operator. The accuracy of co-registration can be impacted by different positions of the patient, and therefore prostate, during the procedures. The patient lays supine in the MRI scanner, while he typically assumes a left lateral decubitus during the TRUS-guided biopsy. Another factor that may impact the co-registration of images is the different obliquity with which MRI and TRUS images are obtained (Figure 2). Transverse MRI is typically acquired on the axial plane of the patient, while TRUS images are more often oblique images of the prostate. In spite of these differences, co-registration is aided by usual anatomical landmarks (such as the urethra), as well as by other distinct findings (such as benign prostatic hyperplasia nodules) that are visible on both images. In the absence of a clear sonographic correlate, however, cognitive targeting can prove very challenging (Figure 3).

### MRI-TRUS fusion targeting

Fusion targeting employs a computer algorithm to map the prostate and regions of interest (*i.e.* targets) from the MRI data and then projects them onto real-time TRUS images. Typically, a radiologist interprets the MRI scan and identifies the target lesions prior to the biopsy.

Multiple systems are available to fuse MRI and TRUS images. Processing involves identifying the boundaries of the prostate and drawing its contours on a reference high-resolution T2-weighted sequence, followed by outlining the targets on this reference sequence. This can be done by the radiologist in advance, even days before the scheduled biopsy, utilizing a planning software that communicates with the proprietary software utilized by the biopsy device. Less commonly, the drawing of the targets is done at the time of biopsy by the physician who will perform the procedure. In that case, if the physician is not a radiologist, the MR images with the abnormalities identified should be available to guide this process.

Most systems require the biopsy operator to first perform a two-dimensional sonographic scan of the prostate, as prescribed by the vendor of the fusion device, which is then post-processed into a three-dimensional (3D) volume. Once the 3D ultrasound image is constructed, the biopsy operator will also contour the gland, as done with the MRI. Co-registration of the MRI and TRUS maps may be rigid (*i.e.* always retain the same shape) or elastic (*i.e.* deformable, in order to account differences in the shape of the gland). Based on experiments on a phantom, these two registration methods perform similarly, except at the edges where rigid registration seems to be more accurate.^27^

The biopsy approach may be transrectal or transperineal. Targeted biopsies are usually done in addition to standard systematic TRUS-guided biopsies. Vendors employ different techniques to track the position of the endorectal transducer, location of the targets on the 3D volumes, and the biopsy needle path. The three most common techniques involve electromagnetic fields and image-based software, which can track freehand sweeps, and mechanical registration, which indirectly gauges transducer position by tracking movements of a mechanical arm on which the transducer must be mounted. While it increases the overall device cost, a potential benefit of the latter method is the mechanical arm’s ability to hold the biopsy device perfectly still, particularly at time of firing, which may improve its accuracy.^28^ However, electromagnetically tracked devices have also been shown to perform well in clinical settings and permit a freehand technique that is more intuitive to most operators.^28^

While the level of operator-dependence of MRI-TRUS fusion targeting is not as great as with cognitive fusion targeting, the operator must nevertheless verify that the images are correctly registered before sampling. Notably, in the previously described phantom study comparing the performance of registration algorithms, the impact of operator experience on registration error was more significant than that of the type of algorithm employed.^27^ Meng et al found a significant institutional learning curve with MRI-TRUS fusion targeting, increasing the cancer detection rate in PI-RADS 4/5 lesions on MRI by 26% over 4 years.^29^ Similar learning curves likely also apply to both the interpreting radiologist and pathologist.^30^

MRI-TRUS fusion targeting therefore still requires significant subspecialty expertise on the part of both the interpreting radiologist and the urology operator, both of whom must have training in compatible targeting systems and must devote time to prostate gland segmentation and target definition.^28^ Moreover, the upfront cost of MRI-TRUS systems may be as high as $300,000 (at the time of writing), in addition to maintenance.^31^

**Figure 4.**
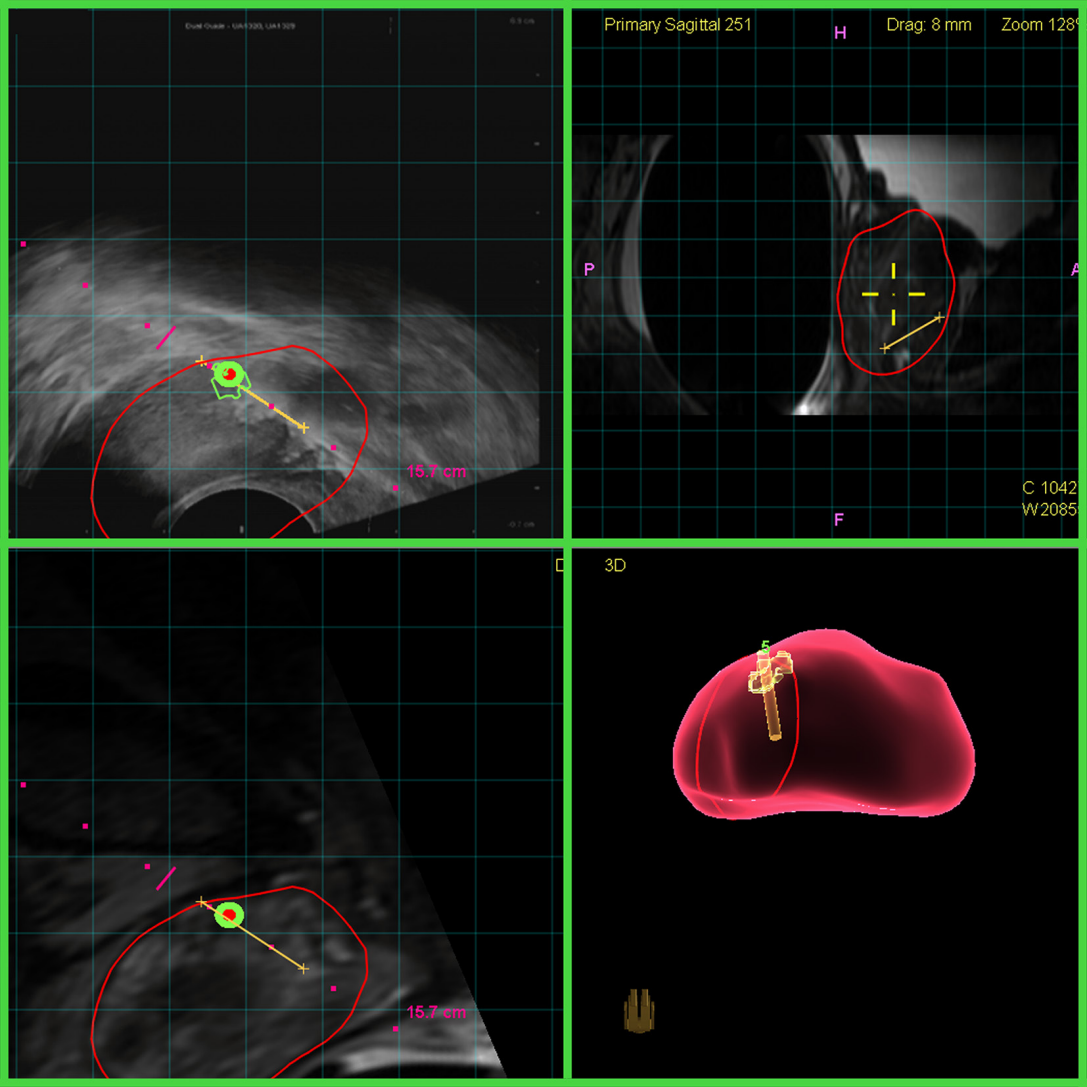
MRI-TRUS fusion targeting. The green and red circles seen in the squares of the left represent the center of the target lesion identified on MRI (bottom left), transposed to the real-time TRUS image (top left). An oblique sagittal reconstruction of the MRI is shown in the top right square. The right bottom square contains the needle tracking map. This allows the successful biopsy of targets such as the one depicted in Figure 3. TRUS,transrectal ultrasound.

In spite of these hurdles, MRI-TRUS fusion targeting is becoming the method of choice for urologists in the United States. While some studies have shown MRI-TRUS fusion biopsies to have an increased cancer detection rate compared to cognitive MRI targeting,^32,33^ even when the cognitive targeting is performed by an experienced provider, the recent randomized controlled FUTURE trial demonstrated no such difference in the detection of overall prostate cancer or of GS ≥3 +4 disease.^34^ One indisputable advantage of MRI-TRUS fusion targeting, however, is that it can record the locations of biopsies performed on the 3D fusion model, allowing them to be re-biopsied at future visits (“tracking”), the important clinical utility of which will be reviewed later (Figure 4).

### In-bore MRI targeting

In-bore targeting, as the name implies, is performed with the patient inside the MR gantry, using an MR-compatible biopsy system mounted to the table. The procedure may be performed using a transrectal or transperineal approach. The position of the biopsy needle introducer and guide (transrectal approach) or the needle insertion location in the template (transperineal approach) is determined using proprietary software. Direct MRI visualization of needle placement into the target lesion allows for high accuracy, needle tracking, and immediate visual confirmation that the desired lesion was sampled. Proponents of this technique also highlight the need for fewer samples to characterize the lesions seen on MRI. Its opponents say it does not completely evaluate the disease status of the patients, as only three or four targets can be sampled per procedure, and tumors that are not clearly visible on MRI will not be detected. Furthermore, in-bore targeting is extremely costly. It not only requires the use of expensive MR-safe equipment, but due to the long amount of magnet time, it affects the workflow of most radiology departments and is uncomfortable for the patient (particularly in closed MR systems). A recent study by Friedl et al showed no significant correlation between operator experience and cancer detection rate, although the biopsy time required and the number of cores taken—both key drivers of cost and patient discomfort—fell significantly between the operators’ first and second year of experience.^35^

### Accuracy of MRI-targeted biopsies

Two meta-analyses evaluating MRI-targeted biopsy techniques versus conventional TRUS-guided biopsy^36,37^ concluded that all MRI-targeted biopsy techniques have a higher rate of clinically significant cancer detection (91% *vs* 76%)^37^ and a lower rate of clinically insignificant cancer detection (44% *vs* 83%)^37^—in other words, lower rates of both underdiagnosis and overdiagnosis.

Additional validation of the use of MRI-targeted biopsy was provided by the prospective PRECISION study, which randomized males with clinically suspected prostate cancer but no prior biopsy to either MRI-targeted biopsy or conventional TRUS-guided biopsy.^38^ In this study, MRI-targeted biopsies could be performed either with cognitive targeting or MRI-TRUS fusion, and either transrectally or transperineally, reflecting the variations in approaches that exist in the community. The study corroborated the findings of the meta-analyses, finding that MRI-targeted biopsy detected significantly more clinically significant cancer, and significantly less insignificant cancer, than conventional TRUS-guided biopsy.^38^

Wegelin et al further analyzed the performance of the individual MRI-targeting techniques both in a metanalysis^36^ and in the randomized controlled FUTURE trial,^34^ both times finding no significant difference in detection of prostate cancer between any of the three MRI-targeting techniques. This argues that the simple use of MRI for target identification is more important than the method of targeting itself. That being said, reproducibility of the biopsy may be highest with MRI-TRUS fusion given its “tracking” capability, of particular import in the setting of AS.

A study of MRI-targeted biopsies by Pahwa et al found that all approaches were more cost-effective than conventional TRUS-guided biopsies, even if a conventional TRUS-guided biopsy was performed when MRI showed no lesions suspicious for cancer.^39^ Although MRI-targeted procedures are individually more costly than conventional TRUS-guided biopsies, MRI tends to detect more clinically significant than insignificant cancers, leading to fewer biopsies, unnecessary treatments and complications, ultimately providing a cost savings.

One of the few remaining areas of controversy is whether or not biopsy-naïve males undergoing MRI-targeted biopsy should undergo any systematic sampling in addition to the targeted sampling of the lesions seen on MRI. Siddiqui et al.’s study, in which the same patients underwent concurrent MRI-targeted and conventional TRUS biopsy, showed that systematic sampling detected GS ≥4 +3 cancers missed by MRI-targeting in 5 out of 542 patients thought to have no cancer based on MRI-targeted biopsy—but that for every 1 such case, 17 additional cases of clinically insignificant cancer were diagnosed: the overdiagnosis problem returned.^40^ More recently, however, Diamand et al found a significant reduction in upgrading at prostatectomy when combining systematic and MRI-targeted biopsy (23.9% *vs* 39.5% with MRI-targeting alone), for only a modest increase in downgrading (12.9% *vs* 9.3%).^14^ With regards to cost-effectiveness, Pahwa et al.’s found that performing systematic biopsy in the event of a negative MRI result remains more cost effective than conventional TRUS biopsy alone, but less cost effective than performing only MRI-targeted biopsy.^39^ Nevertheless, ACR guidelines continue to recommend MRI-targeted biopsy as a supplement to systematic biopsy, rather than a replacement.^41^

### Impact of MRI-targeted biopsy on patient management

While prostate cancer-specific mortality is very low in patients on TRUS-guided biopsy AS protocols— between 0.15 and 1.5% depending on eligibility criteria^42,43^—the number of males who drop AS for therapy is significant: 37%, 50% and 57% at 5, 10 and 15 years in a study published by Tosoian et al.^43^ A biopsy technique that more accurately characterizes disease would improve patient selection and allow for closely monitored intermediate-risk patients to be eligible for AS.

Multiparametric MRI has shown itself to be such a technique. By purposefully oversampling suspicious lesions, MRI-targeted biopsy can improve the detection rate of high-risk foci disease, improving the correlation between the biopsy cores and whole-organ pathology.^14,44^ For example, Hu et al report that a MRI-targeted confirmatory biopsy performed in males thought to be eligible for AS by conventional biopsy upgraded disease 36%.^45^ Similar studies have confirmed this high number, with a range in the literature of 26–42%.^46^

In addition, repeat biopsies using conventional TRUS are insensitive at detecting the progression of a known cancer to higher-grade disease. By contrast, MRI-TRUS fusion biopsies have the ability to precisely track and re-biopsy the same lesion across many visits, providing important longitudinal data on a patient’s prostate cancer. Palapattu et al demonstrated the accuracy of lesion tracking by showing that the same clone of cells could be extracted on both initial and repeat biopsy of a given lesion in 96% of cases.^47^ Clinically, tracking is highly important as it may double the sensitivity for tumor upgrading on repeat biopsy.^48^

Together, the improved correlation with whole-organ pathology and the lesion-tracking capabilities of MRI-targeted biopsy techniques provide more accurate initial and longitudinal grading of disease. This has resulted in a liberalization of AS selection criteria to include intermediate-risk disease: whereas the initial Epstein criteria for AS^49^ excluded any male with GS four pattern, the inclusion of males with low-volume GS 3 + 4 disease is now considered appropriate,^50^ following studies showing that they were no more likely to develop high-risk disease than males with GS 3 + 3 disease.^51^ Nevertheless, intermediate-risk patients should be closely monitored and may undergo further risk-stratification, including on the basis of MRI findings (such as PI-RADS five lesion) or molecular markers.^52^

Taking these technological developments into account, authors such as Elkhoury et al have proposed updated management pathways that make use of MRI-TRUS fusion biopsies and their tracking ability.^46^ Initial biopsies should ideally performed using MRI-TRUS fusion targeting, and confirmatory biopsies should always be performed using MRI-TRUS fusion targeting, with expedited timing for males with GS 3 + 4 disease. If the confirmatory biopsy upgrades the patient’s cancer to GS ≥4 +3, definitive treatment is usually recommended. Otherwise, patients are followed with MRI-TRUS fusion biopsies every 12–24 months with tracking biopsies of the cancerous lesions. If GS 3 + 4 disease is present, MRI-TRUS fusion biopsies are performed every 6–12 months.

### Future directions

Gallium 68-labeled prostate-specific membrane antigen-11 (^68^Ga-PSMA) positron emission tomography (PET) has been shown to be useful in detecting prostate cancer both within the prostate gland^53^ and in recurrent and metastatic disease.^54^ In addition, it has more recently been shown to increase sensitivity for localized cancer when performed in conjunction with multiparametric MRI as ^68^Ga-PSMA PET/MRI. A study by Hicks et al of 32 patients with known prostate cancer ahead of radical prostatectomy found that ^68^Ga-PSMA PET/MRI detected prostate cancer in 97% of patients with the disease *vs* 79% by multiparametric MRI alone, using whole organ pathology as the gold-standard.^55^ Particularly with the increasing prevalence of PET/MR scanners, this modality may provide not only increased sensitivity for prostate cancer, but also a "one-stop shop" in which both localized disease and regional or distant metastases are diagnosed in a single study.

Another promising variation of multiparametric MRI in the diagnosis of prostate cancer is hyperpolarized Carbon 13 MR spectroscopy (^13^C-MRS). Hyperpolarized ^13^C produces >10,000-times greater signal on MRI than ^12^C.^56^ Using MRI, one can thereby generate spectral arrays showing the metabolites of a given hyperpolarized ^13^C-labeled biomolecule. In particular, hyperpolarized ^13^C-labeled pyruvate has been shown to be of particularly utility in the imaging of cancers, including prostate cancer, which are more reliant on anaerobic respiration than normal cells and so metabolize a much greater proportion of pyruvate into lactate, resulting in a high ^13^C-lacate/^13^C-pyruvate ratio. Early human trials of this metabolic MRI have already begun, with promising results.^56^

Notably, data from both PET/MR and ^13^C-MRS are co-registered to the conventional MRI with which they are concurrently acquired. As a result, both of these advances may ultimately serve as new "parameters" in multiparametric MRI and may be used in the same way to identify and target suspicious lesions, with greater sensitivity and prognostic value than is currently possible.

## CONCLUSION

The increasingly wide adoption and standardization of multiparametric MRI, as well as the development of MRI-TRUS fusion techniques, have had a significant impact in the imaging and tissue diagnosis of prostate cancer. As the GS of MRI-TRUS fusion biopsy cores more closely matches that from whole-organ pathology, males with prostate cancer are being risk-stratified more accurately. Suspicious lesions can also now be tracked and biopsied reproducibly over time. In turn, criteria for eligibility for AS are being liberalized and AS management protocols are evolving.
